# Family Caregivers’ Experiences of Preventing Harm to Older People during Hospitalization: A Phenomenographic Study

**DOI:** 10.3390/ijerph192215375

**Published:** 2022-11-21

**Authors:** Chin-Yen Han, Chun-Chih Lin, Li-Chin Chen, Shou-Hsuan Liu, Suzanne Goopy, Wen Chang

**Affiliations:** 1 Department of Nursing, Chang Gung University of Science and Technology, Taoyuan 33303, Taiwan; 2 New Taipei Municipal TuCheng Hospital, Chang Gung Medical Foundation, New Taipei City 236017, Taiwan; 3 Department of Nephrology, Chang Gung Memorial Hospital at Linkou, Taoyuan 33303, Taiwan; 4 Graduate Institute of Clinical Medical Sciences, Chang Gung University, Taoyuan 33303, Taiwan; 5 Usher Institute, University of Edinburgh, Edinburgh EH8 9YL, UK

**Keywords:** preventing harm, family caregivers, integrated care, phenomenography, qualitative study

## Abstract

Hospital admission is associated with a high risk of harm, particularly for older people, and family members play a critical role in providing care. The aim of this study was to explore family caregivers’ experiences in preventing harm to older people during hospitalization. The phenomenographic approach was applied. Thirty family caregivers were asked to describe their experiences of preventing harm to older people. Semi-structured interviews were audiotaped and transcribed. Participants described preventing harm as “essential care”, “an important step toward recovery”, “a load off the mind”, “outcomes of collaboration among caregivers and health professionals”, and “improvement in the quality of life after discharge”. The core theme was to achieve the goal of integrated care for older people. The results can help improve caregiving processes and prevent harm to older people during hospitalizations. They can assist in developing strategies for the delivery of safe care for older people.

## 1. Introduction

The aging population is increasing rapidly worldwide. With the aging population exceeding 16.2% of the total in 2021, Taiwan’s society officially became an aging one. The older population is expected to reach 20% in 2025, making Taiwan a hyper-aged society [1]. Older people constitute the healthcare system’s largest consumer group. Studies in Australia, Britain, Canada, the USA, and Taiwan indicate that people over the age of 65 account for 18–30% of emergency care visits and 34.65% of overall emergency medical expenditure [2,3]. The most frequent users of emergency care are over 75 years of age, and the greater a patient’s age, the greater their medical expenditure [4]. Taiwan’s National Health Insurance records indicate that patients over the age of 65 account for 28.7% of the total medical expenditure, with an average expenditure 3.4 times that of all insured patients. It has been predicted that older people’s emergency care usage and medical expenditure will continue to rise [5,6,7]. The incidents of harm are higher among older people than other age groups [8]. Studies have indicated that occurrences of harm may cause older people to lose function and independence, increasing their caregivers’ burden [9,10]. Thus, preventing harm to older people is crucial.

Incidents of patient harm continue to occur [11]. Patients’ experience of harm in the healthcare system constitutes an international problem. The World Health Organization (WHO) defines patient harm as an incident that results in a patient’s impaired functioning or any deleterious effect arising from, or associated with, plans or actions taken during the provision of healthcare [12]. These include medical, diagnostic, and radiation errors; healthcare associated infections; unsafe surgical procedures and transfusion practices; sepsis; venous thromboembolism; falls, and skin injuries [13,14]. One study developed a framework of events commonly associated with preventable harms in older people during hospitalization. Such common preventable harms are related to skin integrity, mobility, continence, pain, medications, deterioration in cognitive skills, abnormal clinical findings, and medical complications [15]. Similarly, the prevention of patient falls and skill-reducing injuries are the principal goals of the Patient Safety established guidelines by the Ministry of Health and Welfare in Taiwan [16].

Patient harm is one of the top ten causes of disability and death in the world [14]. One in ten patients is harmed while receiving hospital care [17]. Medical errors constitute the third leading cause of death in the USA [18]. Harm is defined as the result of an identifiable cause and a recurrent matter that can be prevented [19]. Approximately 50–80% of harm is preventable. Preventable harm can be defined as an injury which results from treatments, care delivery, or omissions in care; it may result in temporary or permanent disability and even death [19]. Statistical data show that approximately 12% of preventable harm occurrences cause permanent disability or patient death [17,19]. In-patient harm has negative financial outcomes for patients, family members, and the healthcare system [20]. In Australia, preventable harms cost the health sector at least two billion per year [21]. A total of 15% of hospital funds can be attributed to the management of harm events [17]. The loss of income and productivity associated with patient harm is estimated to be trillions of dollars annually, adding to the financial burden of the patient and family caregivers [17]. Therefore, harm prevention can benefit patients, caregivers, and healthcare providers at the individual, organizational, and societal levels.

Taiwanese society has a family-centered culture. Family members must often assume the responsibility of being primary caregivers for older people as part of family ethics [22]. The family caregiver in Taiwan is the person mainly responsible for taking care of a dependent person at home. The informal caregiver is defined as a pre-existing relationship with a person requiring care for a functional dependency due to a health- or aging-related condition [23]. The role of family caregivers in Taiwanese culture is often taken for granted. The family caregiver not only assists in their activities of daily living but is also a key decision-maker for the dependent person [22]. Statistical data from Taiwan’s Ministry of Health and Welfare (2018) show that more than 67% of dependent elderly were taken care of by family members. Women accounted for 60.9% of primary family caregivers, dedicating on average 7.8 years of care time at an average of 11.1 h of care per day [24]. Meanwhile, studies on the benefits of family engagement in care have reported greater medication accuracy and more realistic expectations of patient health beliefs and habits. In addition, families can reorient patients toward taking safety precautions [25].

## 2. Materials and Methods

### 2.1. Rationale and Aims

Efforts to minimize the harm experienced by older people should consider the views of family caregivers. The significance of harm prevention in continuity of care is multifaceted [26,27,28]. The literature emphasizes that family caregivers play an important role in the recovery process and are the main source of support for older people during hospitalization [29,30,31]. However, harm prevention in primary care has rarely been explored from the perspective of older people’s family caregivers, even though understanding their experiences is necessary for improving the quality of care for the elderly. The aim/purpose of the present study was to address this gap by: (1) exploring and describing family caregivers’ experiences; and (2) identifying the concepts that contribute to preventing harm to older people during hospitalization.

The qualitative approach of phenomenography was applied in the study. Phenomenography is a methodology that allows researchers to describe the major features of a phenomenon and the different ways in which a group of people relate to it. It focuses on the phenomenal and experiential aspects of knowledge, emphasizing the common thinking that occurs when people experience a phenomenon. This qualitative design is descriptive, interpretive, and contextual, focusing on people’s descriptions of an experience [32,33]. Phenomenography is also a set of assumptions about humans, science, and how knowledge about people’s ways of experiencing the world can be acquired. It is used to map the qualitatively different ways in which people experience, conceptualize, perceive, and understand various aspects of phenomena and the world around them [32,33].

The results of a phenomenographic study include descriptive categories and the outcome space. The former represents discrete conceptions; they describe similarities and differences in meaning, and reflect the qualitatively different ways in which phenomena are described, analyzed, and understood. The outcome space is the core theme and a diagrammatic representation of the logical relationships between categories describing the phenomenon under investigation [32,33]. This approach allowed the ways in which family caregivers avoid preventable harm to older people to be identified and described. It facilitated the qualitative mapping of how family caregivers experience and understand harm prevention. The advantage of the phenomenographic approach in this study was its focus on family caregivers’ collective understanding of, and relationships with, the phenomenon.

### 2.2. Recruitment, Criteria and Sampling

This study was conducted at a hospital in Taiwan with 3700 inpatient beds. Purposive sampling was used to select participants. The selection criteria for patients were that they: (1) were over 65 years old; (2) had a history of falls, skin injury, pain, or delirium in a previous admission; and (3) had experienced no falls, skin injury, pain, or delirium in their current hospitalization. The selection of the above preventable harms is based on the literature [5]; these harms also commonly occur in Taiwan. Paid caregivers were excluded from the study. In order to ensure selection of a representative group of participants, the recruitment process started with a manual check of the medical computer database of the ward to identify potential patients, after which their family caregivers were approached. The researcher explained the study and invited the family caregiver to participate in a face-to-face interview. Participation was voluntary. Those who met the selection criteria received a Participant Information Sheet outlining the purpose of the study, the voluntary nature of involvement, and an assurance that confidentiality and anonymity would be maintained. Each participant signed a consent form before the interview.

Interviews are the primary method of phenomenographic data collection [32,33]. The literature indicates that the purpose of using interviews in a phenomenographic study is to encourage participants to express their feelings and experiences as much as possible [34]. A semi-structured interview allows a participant to describe their experiences freely while ensuring that the interviewer can clarify the meaning of the words used [34,35]. The interviews in the study focused on family caregivers’ thoughts concerning preventing harm during hospitalization. The interview questions in a phenomenographic study need to be as open-ended as possible in order to remain true to the participant’s thoughts [32,33]. The interviewer began with a simple question, such as “Could you describe how the patient (your father, mother, or other relation) was admitted to the ward?”. This question helped the participants to start talking about their experiences. The researcher then directed the family caregivers to discuss their experiences of preventing harm during the hospitalization as specific harm incidents had previously occurred to the patients. Clarifying questions used included “Could you give me an example?” and “Could you explain that further?”. The clarifying questions helped when the interviewer did not understand participants’ meaning and also encouraged participants to describe their experiences fully [32,33]. The interview guide is attached as an Appendix A.

Phenomenographic studies have shown that a range of possible ways of understanding a phenomenon can be determined when 20 participants are interviewed [32]. In this study, 30 participants were recruited. This number was sufficient to generate rich qualitative data, because the data became saturated, and no new concepts were discovered toward the end of the data collection process. All interviews were audio-recorded and conducted by one researcher (CYH), who was the main interviewer and responsible for the data analysis in the present study. This researcher studied qualitative research during her PhD course, has conducted qualitative research projects as a primary investigator, and has published qualitative research papers. The interviews were conducted from May 2018 to January 2019 and lasted 48–72 min each.

### 2.3. Data Analysis

After each interview, the researcher transcribed the audio-recording and documented the participants’ sentences, pauses, and special terms. The seven steps of phenomenographic data analysis are: familiarization, condensation, comparison, grouping, articulation, labeling, and contrasting [36]. The detailed data analysis strategies of this phenomenographic study, based on the literature [36], are listed below. First, for data familiarization, the researcher read through the transcripts multiple times. The familiarization process enabled the identification of meaningful units from the interview transcripts. Condensation involved the identification of significant descriptions that summarize the understanding of the phenomenon [36]. The researcher identified the important words and statements that summarized the family caregivers’ experiences with, and understanding of, preventing harms. The comparison process involved comparing the identified meaning units in order to find similarities and differences. Meaning units were organized according to agreement before being organized into more specific groups [36]. In the grouping step, the similarities and differences between concepts were recognized. The important statements of family caregivers were highlighted and grouped accordingly [36]. The researcher attempted to explain the participants’ experiences related to each theme in the articulation stage, which was repeated several times until the description of each category reflected the understanding of the phenomenon. The labeling step involved the identification and naming of descriptive categories [36]. Finally, the researcher contrasted the similarities and differences of the findings for each theme [36]. The researcher revisited the interview transcripts and themes several times to establish a diagrammatic representation of the core theme.

The limitation of phenomenographic study is the generalizability of the research outcomes. This study explored family caregivers’ experience on preventing harm to older people in Taiwan; however, this may not necessarily be generalizable to the population as a whole. The data analysis of phenomenographic study derives descriptions from the particular individuals chosen from a certain context [32,33,34]. The participants in the present study were selected on the basis of their appropriateness to the aim of the study. The purposive sampling and the number of participants provided sufficient richness of experiences during data analysis.

### 2.4. Rigor

Trustworthiness in qualitative research means methodological soundness and adequacy. The fundamental outcome of describing a phenomenon is the development of the process, which determines the quality of data in a phenomenographic study [32,37,38]. In a phenomenographic study, moreover, rigor is commonly established by following the principles of faithful description, interpretative awareness, and communicative validity. In order to describe, as faithfully as possible, the family caregivers’ conceptions and experiences of preventing harm, this paper demonstrates how the research team reported and interpreted the participants’ conceptions. Interpretative awareness is enhanced by the rules of bracketing [38], which involves putting aside any researcher bias about the phenomenon. Participants were encouraged to describe the phenomenon based on their own experiences and to structure their explanations in terms of their awareness. Horizontalization was used to ensure that all aspects of the descriptions were treated equally. Communicative validity is relevant to establish data through in-depth, focused dialogues [38]. The phenomenographic study required a second researcher to examine classification of categories and thus establish the reliability of the results [39]. During the data analysis process, quotations from, and descriptions given, by participants were applied to support the generation of the themes. Additionally, to maximize the quality of outcomes, the interpretation of the family caregivers’ experiences in the study was discussed with another researcher (CCL), a qualified and experienced qualitative researcher.

## 3. Results

The participants consisted of 30 family caregivers; 12 male and 18 female, aged 49–76 years. In terms of caregivers’ relationship to the patients, there were 13 husbands/wives (43.3%), 14 sons/daughters/daughters-in-law (46.7%), and three grandsons/granddaughters (10%). The participants are identified as participant one (P1) to participant thirty (P30) in the study. The demographic details of participants are shown in Table 1. The five conceptions of preventing harm to older people identified by family caregivers are as follows: “essential care for patients”, “an important step toward recovery”, “the feeling of a load taken off the mind”, “outcomes of collaboration among family and health professionals”, and “improvement in the quality of life after discharge”. The core theme was “achieving the goal of integrated care for older people and family caregivers”.

### 3.1. The Five Conceptions by the Family Caregivers for Preventing Harm to Elderly Patients

#### 3.1.1. Categories of Description One: Harm Prevention as “Essential Care for Patients”

The family caregivers stated that older people should receive good care while hospitalized, as new problems can cause rapid changes in a patient’s condition. The family caregivers had high expectations of medical care during hospitalization and could not accept the occurrence or impact of harm. A participant mentioned falls as an example.

*My mother was hospitalized several times, and she once fell while going to use the toilet. I absolutely cannot accept it happened… It should be common knowledge that the elderly cannot be allowed to fall*. (P1)

*I think that my father shouldn’t have further unexpected or preventable problems in the hospital. This should be very basic for us*. (P4)

The family caregivers who participated in the study referred to preventing harm as part of their daily routine during hospitalization. Routine care activities, such as changing position, preventing falls, and feeding, are all necessary. Participants explained that these actions are not just the daily routine but also essential care for older people, as shown in the following statements by family caregivers:

*I have been taking care of my husband for years. The process is too much for me, and I always feel tired. He has had falls that resulted in a fracture. That experience of admission was bad. Since then, I have always followed the fall prevention strategies nurses taught me. You can see my husband has made good progress this time*. (P15)

*Being the primary caregiver for my mum, I would do anything for her even it is just repeating and little things… I know to turn the patient every two hours, carefully implementing NG feeding to maintain her good nutrition. It seems to be very basic, but they are also fundamental for preventing harmful situations from happening*. (P3)

#### 3.1.2. Categories of Description Two: Harm Prevention as “an Important Step toward Recovery”

Older people constitute a vulnerable group and face many problems while hospitalized. The interview respondents mentioned that they were chiefly concerned about whether the patients recovered, or their conditions progressed, over the course of hospitalization. They consequently worried about whether the occurrence of any problems or complications might have a negative impact on the patient’s recovery. The family caregivers therefore hoped to avoid this situation and move toward recovery, as described by the following statements:

*Mum is getting better and toward to good outcomes. The doctor said Mum’s progressing well without new problems or complications. I know she would be better and discharged home soon*. (P7)

*Father suffers from chronic diseases for a long time. His skin condition and mobility have been steadily worsening. Fortunately, his recent pneumonia was gradually brought under control… no new problems, which gave me greater confidence in his health condition*. (P9)

*My husband has had dementia and diabetes for a long time. Every time he is hospitalized, new problems occur. I remember that he had incontinence and diaper-induced broken skin. I know he is recovering when his problem got fewer and fewer*. (P14)

#### 3.1.3. Categories of Description Three: Harm Prevention as “the Feeling of a Load Taken off the Mind”

Complicated and unexpected problems may occur during the hospitalization of older people. Considering this, the family caregivers expressed that they felt pressured and experienced challenges during care, including those that they could not control. When a patient’s problems significantly improved or no further problems occurred, the family caregiver felt less pressure.

*Last admission, Mum had many problems… at the beginning of this admission, I cried for days. Now, I worry less because Mum is moving toward better outcomes without complications or new problems*. (P17)

*My husband has been bedridden for years. When he was hospitalized for a longer time before, he had a pressure ulcer that resulted in infection and fever. I was afraid he would die. This time, his condition is stable and no drama happened, and I feel less stress*. (P19)

Family caregivers described preventing harm as crucial because harm events affect family members’ psychological status: Harm to the patient negatively impacts their job, they have to pay admission-related fees, and they may even think about issues related to death. Those psychological stressors for family caregivers disappear when harm prevention is successful for patients during hospitalization, as indicated by the following statements:

*…last admission (mother-in-law) had malnutrition and back skin ulcers. I particularly focused on Dad’s feeding and nutrition. I was so worried about recurrent skin ulcers and other complications from malnutrition. So far, everything is good, and there have been no new problems. Now I feel less worried*. (P8)

*I am thankful for the stable condition this admission. I had a physical and financial burden plus chronic diseases. She was very sick, with different new problems during the previous admission. Many bills need to pay, I had to take time off work, and I was even scared of losing her. When the doctor told me that we can go home tomorrow, I nearly cried*. (P20)

#### 3.1.4. Categories of Description Four: Harm Prevention as “the Outcomes of Collaboration among Family and Health Professionals”

Hospitalized older patients have needs that require attention. It is often necessary for medical professionals to consult with specialists or cooperative care experts. When patients and their family caregivers participate in the care process, they contribute to preventing harm. Team collaboration is needed when family caregivers notice that an older people is at risk.

*My wife is an “old” (frequent user) patient here and had complex problems during the most recent visit. This time, nurses, the nutritionist, and the social worker were working together to take care, and her condition was stabilized. Our entire family pitched in, and we could see the good results as she was getting through this time of difficulty*. (P2)

*I thank you for all the medical and nursing staff taking care of my husband those days. Without anyone and me, he definitely can’t be discharged tomorrow. During hospitalization this time, there were lot of staff from different departments involved*. (P10)

The family caregivers emphasize the significance of working with healthcare team members to prevent harm. They described how, for harm prevention to be effective, the healthcare team members must be involved. The goal of preventing harm must be achieved by coordinating the resources of family caregivers and healthcare professionals, especially as older people have different kinds of problems during hospitalization. One family caregiver explains the experience by stating that:

*The worst scenario he (father-in law) had was falls with a fracture, which ended up with the complications of skin ulcers, pain, delirium, malnutrition, and infection. He was admitted to ICU for a long period of time. Although this time he is not in a critical condition, there are a few specialists and a consultation with a dietician. Additionally, nurses help us to understand all the information we are given and apply it to patient care. So Dad can go home soon*. (P13)

#### 3.1.5. Categories of Description Five: Harm Prevention as “Improvement in the Quality of Their Life after Discharge”

The recovery process and subsequent quality of life for older people may be compromised when harm occurs, making preventable harm an important topic in post-discharge care. Family caregivers expressed that preventing harm during hospitalization had a positive impact on patients’ lives after discharge and enabled better recovery after returning home.

*We are going home on Friday. Unlike the last admission, no drama happened this time. It should be a good start after we go home*. (P12)

*Dad was in poor condition after his previous hospitalization. After discharge following a month of hospitalization, he still had many problems requiring care. Everyone in the family was on the verge of collapse. This time, handling the infection went smoothly … Dad’s complexion improved a lot. We are confident that he will continue to be better after going home, and our lives will get better*. (P16)

Continuity of care is important for older people after they are discharged from hospital. Interruption of continuity of care may cause revisits or readmission. Medical care and quality of life are mutually reinforcing—not just during hospitalization, but also during older people’s post-discharge care and adjustment. The family caregivers described their previous experiences of harm events and how such events have affected their life after discharge home. They talked about how a better condition achieved during the current admission would improve life after being discharged home, as in the following statement:

*I have already had some discharge experiences with my mother-in-law. She had problems of recurrent skin ulcers with pain that required her to revisit the emergency department. That made my husband’s and my life so messy. This time, we have done everything nurse told me we were told by the doctors and nurses to prevent (harm), with good results. We are excited to have my mother-in-law discharged back home*. (P18)

### 3.2. Outcome Space: Preventing Harm to Older People during Hospitalization as Achieving the Goal of Integrated Care for Older People

**Figure 1:**
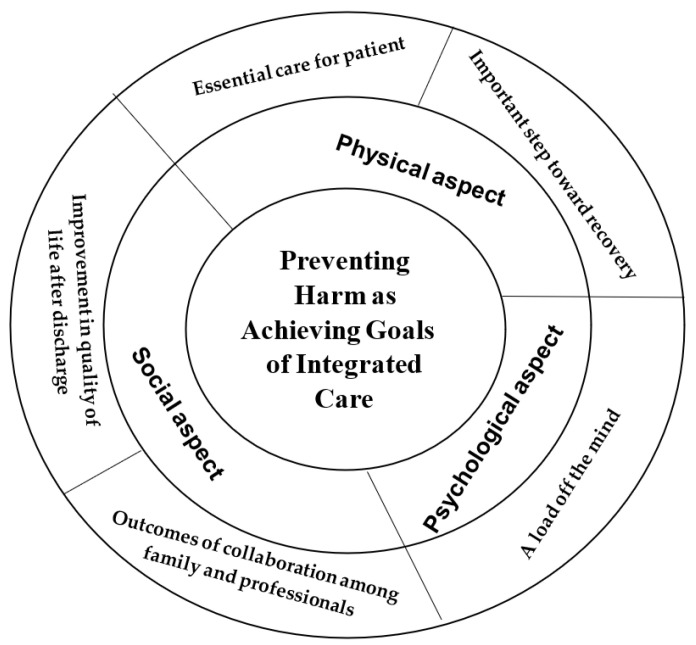
Outcome space--family caregivers’ conceptions of preventing harm to elderly patients during hospitalization.

The outcome space is a representation of the logical relations among the ways harm is prevented, as experienced by family caregivers. In the present study, the outcome space is an arrangement of the five conceptions of harm prevention and consists of three aspects: physical, psychological, and social. These three aspects correspond with the family caregivers’ experiences and understandings of preventing harm to older patients during hospitalization. As mentioned above, harm may cause deterioration of the patient’s physical problems as well as affect the family caregiver’s health status. Family caregivers experienced that preventing harm is essential for older people who need care, and helps them move toward physical recovery. Harm prevention can also decrease the psychological stress of family caregivers caring for older people. They expressed that ensuring harm prevention decreased the psychological pressure of care. The family caregivers perceived that collaboration and cooperation among health professionals and themselves is a crucial process in harm prevention. When the condition of older people improves, both the older people themselves and the family caregivers have a better quality of life after discharge, and the social life of their family caregiver can also progress. Integrated care as a determinant of central relations is described as part of family caregivers’ experiences of being involved in harm prevention for older people during hospitalization. The goal of integrated care represents the central meaning of harm prevention for older people in the caregivers’ understanding and experience. Achieving harm prevention can benefit the patients’ and their caregivers’ physical, psychological, and social health, as shown in Figure 1.

## 4. Discussion

The unique contribution of the present study is its focus on family caregivers’ experiences and understanding of preventing harm to older people during hospitalization. The safety of hospitalized patients is an important issue. In the present study, participating family caregivers revealed that harm prevention is an essential element of care for the older people. When the essential care for preventing harm is well implemented, it facilitates better outcomes for the patient. “Do no harm” is the fundamental element of caregiving [40]. The literature emphasizes that preventing harm critically impacts the mortality rate, subsequent length of rehabilitation, and quality of life of patients [19]. Therefore, strategies for preventing harm should be established at the time of patient admission.

Older people may experience comorbid diseases and require frequent hospitalization. In Taiwan, this may result in numerous stressors among family members, as they are the main caregivers. The literature shows that family caregivers of older people are exposed to physical, psychological, and social burdens [9,10]. Family caregivers experience a decline in quality of life due to a lack of personal time as well as conflicting professional, social, and family demands [41]. The present study revealed that harm prevention during the hospitalization of older people not only allows family caregivers to feel reassured by the improvement of the patient’s condition but also reduces their psychological stress. It is crucial to ensure patient safety by preventing harm.

The results of the present study confirm that prevention of harm during hospitalization is important for older people and family caregivers. It can have positive impacts on patients after discharge. The study demonstrated that experiences of harm may result in extended negative effects upon discharge. This can be more severe for older people [10 Adler, 2018]. Notably, a study in Taiwan demonstrated that family caregivers of older people need to deal with complex, chaotic situations. Shen et al. suggested that the provision of safety care for older people can help patients and family caregivers return to a normal life after discharge [42]. This agrees with the present study’s results related to improvements in quality of life.

Previous research has indicated that views of safety and harm differ between patients, family caregivers, and healthcare providers [43]. Ensuring patient safety in acute care poses challenges for healthcare providers, patients, and their families [19]. Collaboration between family caregivers and healthcare providers is crucial for positive outcomes [44,45]. Family caregivers serve as valuable allies in the process toward recovery. The increased engagement of family caregivers has been identified as an important approach for preventing harm [9,10,11]. As previously mentioned, older people have specific needs, and family caregivers provide crucial support during their medical journeys. Collaboration with family caregivers in care is vital for preventing harm and improving the quality of care after discharge. Patients may find hospital environments intimidating, and familial presence increases feelings of safety. Increasing family involvement is thus imperative for positive patient outcomes and is an important step toward recovery.

In the “Patient Safety Sector” proposed by the Taiwan Executive Yuan, encouraging family caregiver involvement in patient safety is a goal of the safety performance index [16]. This designation emphasizes the importance of family caregivers as advocates of patient safety. Likewise, a US study reported that family involvement is a key factor in harm prevention [25]. A study in Switzerland showed that the integration of family caregivers in delirium-preventing care for hospitalized elderly patients can diminish the negative consequences of hospitalization and reduce harm incidence [46]. The literature suggests that despite various clinical efforts to standardize care delivery, implement systematic safety practices, and provide relevant education, patient harm in hospital settings continues to occur [10]. Increasing the involvement of patient and family caregivers is imperative for patient safety [10,25].

The present study demonstrated that a family caregiver’s experience of preventing harm to an older people involves achieving the goal of integrated care at the physical, psychological, and social levels. With the advancement of medical science and technology, the average life expectancy has increased, and the complexity of today’s medical environment has increased. Many different medical professionals will participate in the multidisciplinary practice of patients, especially in Taiwan’s medical system. The main family caregivers of older people during hospitalization are usually family members. Family caregivers are not medical professionals. It is important to work with medical professionals to provide good integrated care and to prevent any harm that is not caused by the pre-existing disease itself during the patient’s hospitalization.

The present study was conducted at a single hospital in Taiwan. All participants were recruited from two medical wards. Although qualitative research has limited generalizability, the size of the participating group in this study supported the purpose of the research. Nevertheless, the results cannot necessarily be generalized to all family caregivers, which may be a weakness of the study.

## 5. Conclusions

Patient safety is the cornerstone of high-quality health care. Understanding family caregivers’ experiences and conceptions of harm prevention can help healthcare professionals improve the quality of patient care. Hospital administrators and educators should provide appropriate in-service training to ensure that the care needs of patients and family caregivers are met. Further research should be conducted to develop a model of care as well as relevant interventions to reduce harm occurrences. It is necessary to further study family caregivers’ needs from the perspective of outpatient, rehabilitation, and long-term care settings as well as other non-admitted conditions.

## Figures and Tables

**Table 1:** Demographics of participants.

Number	Gender	Age	Relationship to Patient	Patient’s History
P1	F	50	Daughter	Fall and skin injury
P2	M	76	Husband	Malnutrition and skin injury
P3	F	62	Daughter	Skin injury and pain
P4	M	58	Son	Fall and skin injury
P5	M	67	Son	fall and dementia
P6	F	66	Wife	Skin injury
P7	M	56	Son	Dementia and fall
P8	F	50	Daughter-in law	Malnutrition and skin injury
P9	F	65	Daughter	Skin injury and immobility
P10	F	68	Wife	Skin injury
P11	M	68	Husband	Fall and immobility
P12	M	58	Son	Fall and fracture
P13	F	58	Daughter-in law	Fall, skin injury and pain
P14	F	67	Wife	Skin injury and pain
P15	F	71	Wife	Malnutrition and falls
P16	M	51	Son	Malnutrition and skin injury
P17	F	51	Daughter	Skin injury and pain
P18	F	58	Daughter-in law	Skin injury
P19	F	70	Wife	Skin injury
P20	M	69	Husband	Malnutrition and skin injury
P21	M	65	Husband	Skin injury
P22	F	49	Granddaughter	Fall
P23	F	50	Granddaughter	Skin injury
P24	F	64	Wife	Fall and fracture
P25	M	67	Husband	Skin injury
P26	M	50	Grandson	Fall and pain
P27	F	70	Wife	Malnutrition and skin injury
P28	F	69	Wife	Skin injury and Pain
P29	F	60	Daughter-in law	Skin injury
P30	M	51	Son	Malnutrition and skin injury

## Data Availability

The data that support the findings of this study are available on request from the corresponding author, C.-Y.H.

## Appendix A. The Interview Guide

1. Could you describe how the patient (your father, mother, or other relation) was admitted to the ward?
2. Could you describe the caring process related to harm prevention experience to the patient?
3. What did you do to prevent harm during hospitalization?
4. Could you compare current and previous admission in terms of preventing harm?
5. Is there anything you would like to add?
